# Impact of chronic total occlusion lesion length on six-month angiographic and 2-year clinical outcomes

**DOI:** 10.1371/journal.pone.0198571

**Published:** 2018-11-13

**Authors:** Jihun Ahn, Seung-Woon Rha, ByoungGeol Choi, Se Yeon Choi, Jae Kyeong Byun, Ahmed Mashaly, Kareem Abdelshafi, Yoonjee Park, Won Young Jang, Woohyeun Kim, Jah Yeon Choi, EunJin Park, Jin Oh Na, Cheol Ung Choi, EungJu Kim, Chang Gyu Park, Hong Seog Seo, Dong Joo Oh, JinSu Byeon, SangHo Park, HyeYon Yu

**Affiliations:** 1 Department of Internal Medicine, Soonchunhyang University Gumi Hospital, Gumi, Korea; 2 Cardiovascular Center, Korea University Guro Hospital, Seoul, Korea; 3 Department of Medicine, Korea University Graduate School, Seoul, Korea; 4 Cardiovascular Center, Soonchunhyang University Cheonan Hospital, Cheonan, Korea; 5 Department of Nursing, College of Medicine, Soonchunhyang University, Cheonan, Korea; University of Tampere, FINLAND

## Abstract

**Background:**

Successful management of chronic total occlusion (CTO)by percutaneous coronary intervention (PCI) is known to be associated with better clinical outcomes than failed PCI. However, whether angiographic and clinical outcomes following PCI for long CTO lesions differ from those following PCI for short CTO lesions in the drug eluting stent (DES) era remains unknown. We therefore investigated whether CTO lesion length can significantly influence6-month angiographic and 2-year clinical outcomes following successful CTO PCI.

**Methods and results:**

A total of 235 consecutive patients who underwent successful CTO intervention were allocated into either the long or short CTO group according to CTO lesion length. Six-month angiographic and 2-year clinical outcomes were then compared between the 2groups. We found that baseline clinical characteristics were generally similar between the 2 groups. Exceptions were prior PCI, which was more frequent in the long CTO group, and bifurcation lesions, which were more frequent in the short CTO group. Apart from intimal dissection, which was more frequent in the long than short CTO group, in-hospital complications were also similarly frequent between the 2groups. Furthermore, both groups had similar angiographic outcomes at 6 months and clinical outcomes at 2 years. However, the incidence of repeat PCI(predominantly target vessel revascularization),was higher in the long than short CTO group, with our multivariate analysis identifying long CTO as an important predictor of repeat PCI (odds ratio, 4.26;95% confidence interval, 1.53–11.9; p = 0.006).

**Conclusion:**

The safety profile, 6-month angiographic, and 2-year clinical outcomes of CTO PCI were similar between patients with long and short CTO. However, there was a higher incidence of repeat PCI in long CTO patients despite successful PCI with DESs.

## Introduction

The use of drug-eluting stents (DESs) and development of devices for the management of coronary artery stenosis have resulted in the reduction of restenosis and adverse events following intervention[1]. However, despite reductions in restenosis due to DES use, clinical outcomes following percutaneous coronary intervention (PCI) in particular remain strongly affected by baseline lesion characteristics, with greater coronary artery lesion length being associated with poorer prognosis after PCI[2–5].Additionally, despite remarkable progress in the development of DESs, as well as in the development of special devices and techniques for chronic total occlusion (CTO) PCI, procedural success rates in CTO patients are still lower than those in non-CTO patients, with long term outcomes in the former also thought to be affected by baseline lesion characteristics as well as other clinical risk factors[6].It should be noted, however, that lesion characteristics such as the development of collateral vessels differ between CTO and non-CTO patients. As such, risk factors or predictors of adverse intervention-related outcomes in CTO patients may differ from those associated with non-occlusive disease intervention [7].Despite this, there have yet to be any definite findings regarding lesion characteristic-based predictors of CTO PCI outcomes, especially in the DES era.

The aim of this study was therefore to investigate whether CTO lesion length can significantly influence 6-month angiographic and 2-year clinical outcomes following successful CTO PCI. We hypothesized that longer CTO lesions would be associated with more adverse outcomes.

## Materials and methods

### Ethical statement

This study protocol was approved by the institutional review board at Korea University Guro Hospital. All patients or their legal guardians were given a thorough written and verbal explanation of the study procedures before giving written consent for participation in the study.

### Study population

Consecutive CTO patients who underwent successful PCI with DESs from November 2004 to October 2010at the Korea University Guro Hospital Cardiovascular Center in Seoul, South Korea, and who completed 6-month angiographic and 2-year clinical follow-up were enrolled in this study. Patients were included if they were receiving treatment for de novo lesions and excluded if they were being treated for CTO due to in-stent restenosis (ISR). Patients were then allocated into 1 of 2 groups according to lesion length in the target vessel: the long CTO group (angiographic lesion length ≥30mm) or the short CTO group (angiographic lesion length<30mm). CTO lesion length was measured during either was measured during either antegrade or retrograde filling of the distal vessel with simultaneous bilateral dye injection if necessary.

CTO itself was defined as a complete coronary obstruction with a Thrombolysis In Myocardial Infarction (TIMI)flow grade of 0 and an estimated duration of ≥3 months, with or without visible collateral flow[8]. Estimated CTO duration was determined as the time since the last episode of angina symptoms consistent with the location of the occlusion.

### Antiplatelet regimen

Loading doses of clopidogrel (300-600mg) and aspirin (200-300mg) were administered before the index procedure. Following PCI, all patients received aspirin (100mg) and clopidogrel (75mg) as their maintenance dual antiplatelet regimen, with clopidogrel administration being encouraged to continue at least for 1 year. Cilostazol was used as an adjunct to the dual antiplatelet regimen at the treating physician’s discretion, with 200 mg being administered post-loading followed by 100mg twice a day for at least 1month. Other concomitant medications were also prescribed at the treating physician’s discretion.

### PCI procedures

PCI procedures were performed according to current guidelines using standard techniques. Initial antegrade approach with wire escalation was attempted, followed by retrograde approach was performed depend on lesion characteristics and decision of the operator. Various available guidewires were used to cross the CTO lesion and a variety of atheroablative devices and techniques were utilized depending on lesion characteristics. In most cases, simple pre-dilation with a balloon was performed to obtain an adequate luminal diameter, which was necessary to accommodate the unexpanded stent and its delivery system. If necessary, adjunctive balloon dilatation was performed to achieve an optimal outcome. All PCI was performed using DESs, with the DES type used being decided by the operator during the procedure. Procedural success was defined as angiographic diameter stenosis being reduced to less than 30% at final follow-up and a TIMI grade≥2.

### Study endpoints and definition

#### Angiographic outcomes

Quantitative coronary angiographic (QCA) parameters were measured and analyzed before PCI, immediately after PCI, and 6months after the index procedure. This was done at the Korea University Guro Hospital Cardiovascular Center. The primary angiographic endpoint was binary restenosis incidence at 6–12 months. Secondary angiographic endpoints were ISR, mean diameter stenosis, minimal luminal diameter, and late loss (LL) at 6 months after the index procedure.

#### Clinical outcomes

Two years after the index PCI, follow-up data were collected by a face-to-face interview at an out-patient clinic, review of patient medical records, and/or telephone contact with patients. The primary clinical endpoint was the occurrence of a major adverse cardiovascular event (MACE), which was defined as the composite of total death, recurrent myocardial infarction (MI), and revascularization including PCI and coronary artery bypass graft (CABG). Secondary endpoints included death, MI, repeat PCI, target lesion revascularization (TLR), target vessel revascularization (TVR), and non-TVR. Deaths were considered as cardiacun less non-cardiac death could be confirmed. MACEs were categorized as either TLR-MACE or TVR-MACE, which were defined as the composite of cardiac death, MI, and TLR or TVR, respectively. Q-wave MI was defined as the development of new pathological Q-waves in at least 2contiguous leads due to MI with or without an elevated creatinine kinase-MB fraction level.

### Statistical analysis

For continuous variables, between-group differences were evaluated using Student’s t-test, with data expressed as mean ± standard deviation. Categorical variables were instead compared between groups using either the χ2 or Fisher’s exact test as appropriate, and are expressed as counts and percentages. Logistic regression model analysis was carried out to estimate the risk of angiographic and clinical follow-up events with adjustment for risk factors such as sex, age, hypertension, diabetes mellitus, chronic kidney disease, prior PCI, and bifurcation lesions. For all analyses, a 2-sided p-value of<0.05 was considered statistically significant. All statistical analyses were performed using SPSS 20 (IBM SPSS; Chicago, IL, USA).

## Results

### Baseline clinical, angiographic, and procedural characteristics

A total of 235CTO patients who underwent PCI using DESs were enrolled in this study. Of these, 159 patients were allocated to the long CTO group and 76 patients were allocated to the short CTO group. Baseline clinical and angiographic characteristics are summarized in Table 1.

**Table 1:** Baseline clinical, angiographic and proceduralCharacteristics.

Baseline characteristics, N (%)	Long CTO (N = 159)	Short CTO (N = 76)	P-value
**Clinical characteristics**			
**Male, sex**	119 (74.8)	56 (73.6)	0.848
**Age (years)**	60.94 ± 11.19	62.10 ± 10.78	0.446
**Left ventricular ejection fraction(%)**	49.24 ± 11.49	52.17 ± 11.18	0.076
**Prior myocardial infarction**	28 (17.6)	5 (6.5)	0.022
**Prior PCI**	40 (25.1)	10 (13.1)	0.035
**Prior CABG**	3 (1.8)	1 (1.3)	0.751
**Hypertension**	101 (63.5)	46 (60.5)	0.657
**Diabetes**	60 (37.7)	26 (34.2)	0.599
**Dyslipidemia**	49 (30.8)	18 (23.6)	0.257
**Cerebrovascular accident**	13 (8.1)	5 (6.5)	0.666
**Peripheral artery disease**	9 (5.6)	2 (2.6)	0.303
**Chronic Kidney disease**	11 (6.9)	3 (3.9)	0.368
**Smoker**	88 (55.3)	38 (50)	0.442
**Angiographic and procedural characteristics**			
**Treated vessel per Patient**	1.949 ± 0.825	1.855 ± 0.795	0.401
**Treated CTO vessel per Patient**	1.075 ± 0.264	1.026 ± 0.161	0.137
**Multi-vessel disease**	101 (63.5)	46 (60.5)	0.657
**Multi-vessel CTO**	12 (7.5)	2 (2.6)	0.136
**CTO Lesion site**			
**Left anterior descending artery**	74 (46.5)	33 (43.4)	0.653
**Left circumflex coronary artery**	31 (19.4)	23 (30.2)	0.066
**Right coronary artery**	66 (41.5)	22 (28.9)	0.062
**Stented CTO**	147 (92.4)	73 (96)	0.291
**LM disease**	8 (5)	2 (2.6)	0.393
**Bifurcation lesion**	37 (23.2)	29 (38.1)	0.017
**Total Lesion Length (mm)**	55.71 ± 27.16	21.56 ± 5.088	< 0.001
**Vessel Size (mm)**	3.114 ± 0.438	2.920 ± 0.423	<0.001
**Minimal lumen diameter(mm)**	2.726 ± 0.520	2.422 ± 0.679	<0.001
**Acute gain (mm)**	2.726 ± 0.520	2.422 ± 0.679	<0.001
**STENT diameter (mm)**	2.781 ± 0.361	2.861 ± 0.376	0.090
**STENT length (mm)**	29.83 ± 5.690	24.25 ± 5.626	< 0.001
**Max Inflation (atm)**	14.40 ± 3.256	12.75 ± 3.178	<0.001
**IVUS Guided PCI**	20 (12.5)	12 (15.7)	0.502
**Used stents on CTO lesion**	3.283 ± 0.948	2.986 ± 0.720	< 0.05
**Used stent per Patient**	3.962 ± 1.391	3.644 ± 1.174	0.069
**Used stents (CTO lesion)**			
**Sirolimus eluting stent (SES)**	43 (27)	13 (17.1)	0.094
**Paclitaxel eluting stent (PES)**	91 (57.2)	22 (28.9)	< 0.001
**Zotarolimus eluting stent (ZES)**	30 (18.8)	30 (39.4)	< 0.001
**Endeavor sprint**	15 (9.4)	18 (23.6)	0.003
**Endeavor resolute**	15 (9.4)	12 (15.7)	0.153
**Everolimus eluting stent (EES)**	14 (8.8)	13 (17.1)	0.062
**Promus &Xience**	13 (8.1)	13 (17.1)	0.041
**Promus Element**	3 (1.8)	0 (0)	0.228
**Discharge medication**			
**Aspirin**	159 (100.0)	76 (100.0)	ns
**Clopidogrel**	159 (100.0)	75 (98.7)	0.340
**Cilostazol**	93 (58.4)	38 (50.0)	0.265
**Triflusal**	8 (5.0)	3 (3.9)	0.756
**Sarpogrelate**	0 (0.0)	1 (1.3)	0.340
**Statin**	114 (71.7)	53 (69.7)	0.733

CTO: chronic total occlusion; PCI: percuataneous coronary intervention; CABG; coronary artery bypass graft; iVUS: Intravascular ultrasound.

The baseline clinical and angiographic characteristics were similar between the two groups except prior PCI(25.1% vs. 13.1%, p < 0.05) and prior MI (17.6% vs. 6.5%, p < 0.05)were more frequent in the long CTO group whereas bifurcation lesion (23.2% vs. 38.1%, p < 0.05) was more frequent in the short CTO group. Treated vessel size and stent diameter were smaller in the short CTO group.

### Clinical outcomes

The incidence of procedural and in-hospital complications was similar between the 2groups (Table 2).However, significant intimal dissection was more frequent in the long than short CTO group.

**Table 2:** Procedural complications and in-hospital clinical outcomes.

	Long CTO (N = 159)	Short CTO (N = 76)	P-value
**Procedural complications, N (%)**			
**Perforation**	5 (3.1)	0 (0)	0.118
**Dissection**	34 (21.3)	8 (10.5)	0.042
**No reflow**	5 (3.1)	1 (1.3)	0.405
**Any hematoma(<4cm)**	1 (0.6)	1 (1.3)	0.591
**Major hematoma(>4cm)**	6 (3.7)	5 (6.5)	0.340
**Acute thrombosis**	0 (0)	1 (1.3)	0.147
**Acute renal failure**	1 (0.6)	1 (1.3)	0.591
**Cerebrovascular accident**			
**In-hospital clinical outcomes, N (%)**			
**Mortality**	2 (1.2)	2 (2.6)	0.446
**Cardiac death**	1 (0.6)	2 (2.6)	0.200
**Non-cardiac death**	1 (0.6)	0 (0)	0.488

Regarding clinical outcomes at 2years(Table 3), the incidence of adverse clinical events (expressed as long CTO group vs. short CTO group) including total death (3.1% vs. 5.2%, p = 0.43), cardiac death (1.2% vs. 2.6%, p = 0.45),MI (1.8% vs. 2.6%, p = 0.71), and MACEs(21.3% vs. 13.1%, p = 0.13) was not significantly different between groups. On the other hand, the incidence of repeat PCI (18.8% vs. 7.8%, p <0.05), which was predominantly TVR (16.3% vs. 6.5%, p <0.05) was higher in the long CTO group.

**Table 3:** Two-year clinical outcomes.

Variables, N (%)	Long CTO (N = 159)	Short CTO (N = 76)	P-value
**Mortality**	5 (3.1)	4 (5.2)	0.428
**Cardiac death**	2 (1.2)	2 (2.6)	0.446
**Non-cardiac death**	3 (1.8)	2 (2.6)	0.711
**Any Myocardial infarction**	3 (1.8)	2 (2.6)	0.711
**Q wave**	3 (1.8)	2 (2.6)	0.711
**Repeat PCI**	30 (18.8)	6 (7.8)	0.028
**TLR**	22 (13.8)	5 (6.5)	0.102
**TVR**	26 (16.3)	5 (6.5)	0.038
**Non TVR**	4 (2.5)	0 (0)	0.163
**All MACE**	34 (21.3)	10 (13.1)	0.130
**TLR MACE**	23 (14.4)	7 (9.2)	0.258
**TVR MACE**	30 (18.8)	10 (13.1)	0.275

PCI: percuataneous coronary intervention; TLR: target lesion revascularization; TVR: target vessel revascularization; MACE; major adverse cardiac event.

Finally, Table 4 shows the results of the logistic regression model analysis performed to estimate the risk of repeat PCI and TVR. Long CTO was found to be an independent predictor of both repeat PCI (odds ratio [OR], 4.26;95% confidence interval [CI], 1.53–11.9; p <0.05) and TVR (OR,4.23;95% CI, 1.52–11.8; p <0.05).

**Table 4:** Multivariate analysis of risk factor for repeat PCI and TVR.

Variable	OR	95% C.I	P-value
**Repeat PCI**			
**Long CTO**	4.26	1.53–11.9	< 0.05
**Male, sex**	0.49	0.19–1.27	0.146
**Age**	0.98	0.94–1.02	0.490
**Hypertension**	0.67	0.30–1.48	0.334
**Diabetes**	0.67	0.29–1.56	0.362
**Chronic kidney disease**	2.00	0.49–8.13	0.332
**Prior percutaneous coronary intervention**	1.00	0.40–2.50	0.990
**Bifurcation lesion**	2.15	0.94–4.95	0.070
**TVR**			
**Long CTO**	4.24	1.52–11.84	< 0.05
**Male, sex**	0.48	0.18–1.27	0.143
**Age**	0.98	0.94–1.02	0.477
**Hypertension**	0.67	0.30–1.48	0.329
**Diabetes**	0.68	0.29–1.61	0.389
**Chronic kidney disease**	2.24	0.40–12.34	0.353
**Prior percutaneous coronary intervention**	0.72	0.03–13.43	0.826
**Bifurcation lesion**	1.02	0.40–2.55	0.068

CTO: chronic total occlusion; PCI: percuataneous coronary intervention; TVR: target vessel revascularization

### Angiographic outcomes

Angiographic follow-up data at 6–12 months was obtained in 99 of the 159 long CTO group patients (62%),and in 45 of the 76 short CTO group patients (59%) (Table 5).

**Table 5:** Six-month angiographic outcomes.

Variables, N (%)	Long CTO (N = 99)	Short (N = 45)	P-value
**In-stent restenosis**	33 (33.3)	12 (26.6)	0.423
**Binary restenosis**	19 (19.1)	4 (8.8)	0.117
**Diameter stenosis**	28.8 ± 27.15	25.39 ± 20.07	0.422
**Minimal lumen diameter(mm)**	2.159 ± 0.852	2.129 ± 0.669	0.807
**Late Loss (mm)**	0.652 ± 0.822	0.461 ± 0.564	0.134

Angiographic outcomes (expressed as long CTO group vs. short CTO group) including ISR incidence (33.3% vs. 26.6%, p = 0.42), binary restenosis (19.1% vs. 8.8%, p = 0.12), and LL (0.65 ± 0.82 mm vs. 0.46 ± 0.56 mm, p = 0.13), did not significantly differ between groups.

## Discussion

This study has 4main findings. First, the incidence of prior PCI and history of MI were more frequent in the long than short CTO group. Second, except for intimal dissection, which was more frequent in the long than short CTO group, the incidence of in-hospital complications was similar between the 2 groups. Third, regardless of CTO lesion length, both long and short CTO groups had similar major clinical outcomes at the 2-year follow-up, including the incidence of cardiac death, MI, and MACEs. The exception was repeat PCI, which was predominantly TVR, being more frequent in the long than short CTO group. Fourth, the long and short CTO groups showed similar angiographic outcomes at 6 months.

Previous DES era studies have reported that successful CTO PCI is not only associated with improved clinical outcomes such as lower cardiac mortality, but may also be a predictor of reduced CABG rates [9–12]. Furthermore, with increasing experience and technical improvement, as well as developments in stent technology, the procedural success rate of CTO PCI has reached as high as 90%[13, 14].The procedural success rate in our study was similar at 85%.Due to these advancements, attempting to improve success rates and resolving technical problems are no longer treated as major issues in CTO intervention. Despite this, clinical and angiographic outcomes following CTO intervention remain worse than those following intervention for non-CTO disease[15–17]. Furthermore, undergoing treatment for non-CTO disease and lesion length in coronary artery disease are major predictors of MACEs and restenosis after stent implantation with DESs [18–20].Predictors of CTO PCI outcomes therefore still need to be better understood.

At the start of our investigation into the impact of CTO lesion length on clinical outcomes following successful PCI, we hypothesized after careful consideration that adverse clinical outcomes, including MACEs and adverse angiographic outcomes, would be more frequent in the long than short CTO group. There were 2main reasons for this. First, the long CTO group had worse clinical risk factors for adverse clinical outcomes, such as history of prior MI and PCI[21, 22], than the short CTO group. Second, the use of newer generation DESs was dominant in the short CTO group, whereas the paclitaxel-eluting stent was frequently used in the long CTO group. As there is a large amount of data suggesting that newer generation DESs are associated with superior cardiovascular outcomes following PCI[23, 24], we predicted that outcomes would be worse with increased CTO lesion length.

Contrary to our hypothesis, we found that apart from intimal dissection, procedural complications were similar between the two groups. Our finding of more frequent intimal dissection in the long than short CTO group suggests that recanalization procedures were more complex and aggressive in the former. Furthermore, although CTO guide wire passage into a false channel would have been more frequent in the long than short CTO group, in-hospital outcomes were not different between the 2 groups. These results provide support for the procedural safety and efficacy of CTO intervention in both groups during the hospitalization period. Additionally, the incidence of mortality including cardiac death, MI, and MACEs did not differ between the 2groups, with only repeat PCI and TVR being more frequent in the long than short CTO group. Despite long CTO being an important independent predictor of repeat PCI and TVR in our multivariate analysis, our overall 2-year major clinical outcome results suggest that PCI for long CTO lesions with DESs is as safe as CTO PCI for short CTO lesions.

Considering these results, it can be seen that the higher incidence of TVR in the long CTO group did not translate into individual critical hard endpoints such as mortality and cardiac death. This discrepancy between hard endpoints and revascularization incidence in the treatment of CTO has been reported previously in the j-Cypher Registry[16]. Although it is difficult to determine the exact mechanism by which this discrepancy may occur, the presence of lower myocardial viability and previously developed collateral channels may play an important role[25, 26]. It is possible that these factors could lessen the harmful physiologic effect of target vessel restenosis in patients with long CTO lesions.

Regarding angiographic outcomes in the present study, 6-monthangiographic outcomes were statistically similar between the 2 groups. However, there was a trend toward greater incidence of adverse outcomes such as binary restenosis in the long than short CTO group. It should be noted that the binary restenosis rate of 8.8% in the short CTO group is similar to the 9.9% rate reported by Hoye et al. [27]. Thus, considering our small patient population and the short follow-up period, we suggest that the incidence of binary restenosis would have been significantly higher in the long CTO group if follow-up had continued for longer. The higher incidences of repeat PCI and TVR during our2-year follow-up period support this hypothesis. Finally, though vessel size was smaller in the short than long CTO group, both groups had large enough vessel diameters based on a previous small vessel diameter standard [1, 28–30]. Thus, this between-group difference in vessel diameter did not significantly affect angiographic and clinical outcomes.

Although our study successfully investigated a real-world cohort of CTO patients who were evaluated prospectively, it does have some limitations. First, the present study was not a randomized study and patients were drawn from a single-center registry. Furthermore, baseline clinical, lesion, and procedural characteristics lightly differed between the 2 groups. In particular, while newer generation DESs were more frequently used in the short CTO group, first generation stents were more frequently used in the long CTO group. Thus, despite strict statistical adjustment, there could still be unmeasured confounders and analytical bias. Second, follow-up coronary angiography was not routinely performed in all patients. Whether follow-up angiography was performed mainly depended on whether the patient displayed ischemic symptoms or whether physicians deemed it necessary regardless of patient symptoms. Since well-developed collaterals and myocardial viability could influence ischemic symptoms, some confounding factors could not be avoided. Third, our study’s sample size was relatively small, and although we did not find between-group differences in angiographic outcomes, this may have been because our study did not have sufficient power to detect any differences. Fourth, lesion length was mostly measured angiographically. Various other tests such as intravascular ultrasound or optical coherence tomography are necessary for more precise measurement of CTO length. Finally, the 2-year follow-up period was not long enough to evaluate long-term safety and efficacy. Therefore, a long-term study with a larger number of patients is required for more definitive results.

## Conclusions

The safety profile, mid-term angiographic, and long-term clinical outcomes of CTO PCI were similar between patients with long and short CTO lesions. However, there was a higher incidence of repeat PCI in patients with long CTO lesions despite DES implantation. A long-term clinical study with a larger study population is necessary for more conclusive results.
